# On the Use of Muriate of Ammonia in Cynanche Tonsillaris

**Published:** 1852-07

**Authors:** A. B. McKay

**Affiliations:** Green Bush, Illinois


ART. V.—*On the Use of Muriate of Ammonia in Cynanche Tonsillaris*.
By A. B. McKay, Green Bush, Illinois.

Although this disease is rarely fatal, yet it is one of the most distressing the human family is subject to. It is this which has induced me to lay before the profession a few remarks on a remedy which I have used with complete success in many cases of this disease; in fact, in every case it has succeeded, in which I have used it. The remedy is *muriate of ammonia*, or *sal ammoniac*.

My usual method of administering it is as follows:

℞ Ammon. Mur. ʒiii.

Tinc. Opii, fʒi.

Aceti, f℥iv.

Misce.

As soon as the vinegar is well saturated with the ammonia, I let the patient use it freely and often, as a gargle. In the absence of vinegar and laudanum, I have powdered the ammonia and blown it on the affected parts. This remedy has succeeded so perfectly in my hands, that I am almost inclined to think it a specific in this disease. I use it with equal success in all stages.

May 14, 1852.

